# Determination of phthalates in particulate matter and gaseous phase emitted in indoor air of offices

**DOI:** 10.1007/s11356-020-10195-3

**Published:** 2020-09-22

**Authors:** Małgorzata Szewczyńska, Elżbieta Dobrzyńska, Małgorzata Pośniak

**Affiliations:** Department of Chemical Aerosol and Biological Hazards, Central Institute for Labour Protection - National Research Institute, Czerniakowska 16, 00-701 Warsaw, Poland

**Keywords:** Phthalate esters, Inhalable fraction, Respirable fraction, Particle size distribution, Indoor air, Office rooms

## Abstract

Phthalate esters (PAEs) are endocrine disrupters and can disrupt the functioning of different hormones, causing adverse effects on human health. Due to the potential exposure to phthalates in office rooms, their concentrations in the air of these premises after their renovation and furnishing were determined. The aim of the study was to determine the content of these compounds in the gas phase and adsorbed on the particles. Thus, the combined sampler with filters and adsorption tube was used for air sampling. Samples were analyzed by GC-MS. The gas fraction was dominated by dimethyl phthalate (DMP), diethyl phthalate (DEP), and the inhalable fraction by dibutyl phthalate (DBP) and 2-(diethylhexyl) phthalate (DEHP). The total concentration of phthalates in the respirable fraction in the furnished rooms was as much as 92% of the phthalates determined in the inhalable fraction. In the rooms immediately after renovation and those arranged and used by employees for 7 months, their concentration in the respirable fraction did not exceed 25% of the phthalates in the inhalable fraction. Phthalate concentration in the renovated rooms after 7 months of their usage dropped by 84% in relation to PAEs concentration in newly arranged rooms and by 68% in relation to the phthalate concentration in empty rooms.

## Introduction

Phthalate esters (PAEs) cause numerous health disorders, especially in the period of prenatal development and childhood, but also in adults. In men, they contribute to testicular stagnation, underdevelopment of genital organs at birth, reduction of sperm count, benign testicular tumors (later in life), or lowering testosterone levels in blood. In the case of women, phthalates cause premature breast adolescence, sexual cycle disorders, and discontinuation of ovulation.

The health effects of exposure to phthalates also include disorders of the central nervous system and an increased risk of autism. These disorders result mainly from the destructive effect of 2-(diethylhexyl) phthalate (DEHP) on the brain tissue. In recent years, scientific research has provided more evidence of the effects of various allergies and inflammatory reactions to phthalates. It has been shown that allergy and asthma children’s homes have significantly higher concentrations of DEPH, benzyl butyl phthalate (BBP), and di-n-octyl phthalate (DnOP) in house dust than in the samples collected in healthy children’s homes (Jurewicz and Hanke 2011; Oie et al. 1997).

Phthalates are a group of chemicals that are present in building materials and in a large number of retail products such as solvents and packaging. They are also the basis for phthalic paints and varnishes, adhesives, and certain laminates and are used as plasticizers in PVC flooring materials. They can be emitted to the environment in new or newly renovated rooms. The health effects of inhalation of aerosols are related to their physicochemical and biological properties. These, in turn, affect the respiratory tract of the particles and their interaction with cells and tanks at the deposition site.

PAEs as plasticizers are not bound to polymers; therefore, they are easily released to pollute the air in the working and living environment. Due to their physicochemical properties, they can be present in the indoor air both in particle phase with different size distribution and in a vapor phase (Hines et al. 2010).

Despite the common occurrence of phthalates in the indoor air in factories, offices, healthcare facilities, school, kindergartens, as well as in private homes, they have not been sufficiently characterized and recognized. The studies of the latest 10 years on the assessment of exposure to these substances come primarily from Asian countries (Chen et al. 2018; Song et al. 2015; Wang et al. 2015; Zhang et al. 2014). The results of occupational exposure assessment to several phthalates in gaseous and particle fraction during plastics and rubber processing were also previously published by the authors (Szewczyńska et al. 2019). With the latest research on phthalates in different fractions, interesting results have been published by Chinese scientists. Phthalate esters in the particulate matter PM10 and PM2.5 fraction were studied in thirteen homes in Tianjin (Zhang et al. 2014). Dibutyl phthalate (DBP) and DEHP were the most common in these fractions, while DBP and dimethyl phthalate (DMP) were predominant in gas and molecular phase, with the mean concentrations of 573 and 368 ng/m^3^, respectively. In the study of Sun et al. (2017), 410 dust samples from children’s bedrooms in Tijanjin and Cangzhou were analyzed. Six PAEs—diethyl phthalate (DEP), diisobutyl phthalate (DiBP), DBP, BBP, DEHP, and diisononyl phthalate (DiNP)—were found in these samples. Their average values in 1 g of dust were 0.31 μg, 16.4 μg, 42.6 μg, 0.11 μg, 127.1 μg, and 0.28 μg, respectively. Both gaseous and particulate phases of phthalates in office air have been studied by Song et al. (2015). The common PAEs in this case were DEHP, DBP, and DEP, and the total phthalates concentration (sum of gas and particulate phase) were 4748.24 ng/m^3^. Concentrations of phthalates determined as particulates constituted about 40% of phthalates determined jointly in the gas phase and in the phase of particles. Phthalates in PM2.5 fraction constituted 72.6% of total particulate phase.

To estimate the exposure to PAEs in both the gaseous and particulate phases, measurements were also performed in Chinese hospitals (Wang et al. 2015). The highest concentration (24.19 μg/m^3^) was determined in the hospital pharmacies, which was 1.54 times higher than that of newly decorated houses. DEP, DBP, BBP, and DEHP constituted above 80% of all six phthalates investigated. The PAEs in particulates fraction were about 2.1 times smaller than in the gas phase.

PAEs in airborne particles from homes, offices, kindergartens, and public places were investigated in Beijing (Wang et al. 2017). DiBP, DnBP, and DEHP were determined in each of the investigated environments, with detection frequencies greater than 90% in homes, while DEP was detected only in homes in 42% of the investigated places.

Airborne phthalates measurements were carried out also in Singapore’s Child Care Center (Jia et al. 2019). DBP, DEP, and DEHP were present in greater abundance in indoor air than DMP and BBP. Median indoor air concentrations of gas and particle phases of DBP, DEHP, and DEP were 2.53 μg/m^3^, 1.33 μg/m^3^, and 1.20 μg/m^3^, respectively. The median indoor air concentrations of DMP and BBP were one order of magnitude lower, which were 0.27 μg/m^3^ (range 0.11–0.57 μg/m^3^) and 0.11 μg/m^3^ (range 0.060–0.17 μg/m^3^), respectively. For the chemical composition, the content of DBP was equaled to 29–61% of the total indoor phthalates, which was followed by DEHP (13–34%) and DEP (14–32%).

In indoor air of homes in Tianjin, six phthalate esters in PM10 and PM2.5 fractions were collected. The results showed that the concentrations of 6 PAEs in indoor PM10 were in the range of 13.88–1591.27 ng/m^3^ and PM2.5 fraction was in the range of 7.26–1244.18 ng/m^3^. In their study, DBP was the most common phthalate followed by DEHP (Chen et al. 2018).

Since PAEs are endocrine disrupters and can disrupt the functioning of many different hormones, they can cause many adverse effects on human health. These may include fertility and reproductive disorders in women and men, premature puberty of girls and boys, increased risk of neurological disorders, increased risk of developing allergies, and increased risk of developing certain cancers (Adibi et al. 2003; Colón et al. 2000; Foster 2001; Lovekamp-Swan and Davis 2003). It is therefore important to monitor these substances in the various environments (Dobrzynska 2016; Hauser et al. 2006). Because of the time that employees spend in offices and the equipment they use there, this environment is an important source of exposure to PAEs. It should be emphasized that many people spend at least 8 h a day at work. There is also a large amount of plastic material and electronic equipment in the office rooms, from which phthalates can be emitted into the air and thus their concentrations can be found at significant levels (Song et al. 2015).

According to the literature review (Bu et al. 2019; Chen et al. 2018; Chi et al. 2017; Pei et al. 2013), there are not many studies on the determination of phthalates separately in the gaseous phase and different fractions of airborne particles in the office rooms and other environments. Therefore, the aim of this study was not only to determine the phthalates in air samples taken in the office rooms after their renovation but also to compare the concentration levels of these substances immediately after the renovation of the rooms, then after equipping them with office furniture, and finally after a certain period of using the rooms by the employees. The research presented in this paper focuses on the determination of phthalates occurring in the office rooms in the gas fraction and in the particles with different aerodynamic diameters ranging from 100 to 4 μm. This range of aerodynamic diameter of particles is related to the place of its deposition in the respiratory system and corresponds to the inhalable and respirable fractions as it was previously described by the authors (Szewczyńska et al. 2019). The inhalable fraction is defined as a mass fraction of total airborne particles which is inhaled through the nose and mouth and the respirable fraction as a mass fraction of inhaled particles which penetrate to the unciliated airways (ISO 7708:^1995^).

## Materials and methods

### Chemicals and materials

All chemicals and solvents used for extraction and gas chromatography (GC) analysis were of HPLC grade. Phthalate Standard mixtures EPA Method 8061A Phthalate Esters Mixture were purchased from LCG Standards as stock solutions in hexane:acetone (80:20). The concentration of each phthalate was 1.0 μg/mL, respectively.

### Sampling sites and conditions

Air samples were collected in 5 renovated office rooms in Warsaw in the period from January to October. Twenty-four hours before the sampling and during the measurements, all the doors and windows were closed. The first measurements were carried out in five rooms immediately after the renovation, which focused on painting walls, replacing windows and laying new floor coverings. Subsequent measurements in selected office rooms were carried out after the furniture assembly had been completed. The rooms had new desks, wardrobes, chairs, electronic equipment, computers, and printers. A third series of tests was carried out in the renovated and furnished offices where the officials worked for 7 months. Employees used printers and computers, used air conditioning, and opened windows at the same time. Therefore, outdoor air samples were additionally taken to eliminate the effect of phthalates from the outside on indoor measurements. The concentrations of phthalates in the external samples were below the limit of detection.

Since phthalates can be present in the environment in both the particulate fraction and the gas phase, a combined sampler with IOM (SKC, Inc., USA) and adsorption tube was used for air sampling. IOM sampler, developed at the Institute of Occupational Medicine (IOM) in Scotland, was described in the previous work of the authors (Szewczyńska et al. 2019) more in details. The front of the IOM cassette was equipped with MultiDust Foam Disc (polyurethane foam), and 25-mm thick glass fiber filter (Whatman) was placed in the back of the cassette. The absorption tube filled with Amberlite XAD-2 resin (Sigma Aldrich, USA) has been connected to IOM sampler in order to ensure the collection of PAEs present in both gaseous and particulate fraction. By flowing through the air sampler, the fine particles that are respirable fractions pass through the polyurethane foam and stop at the filter. The inhaled fractions are the particles retained on the PUF foam and the filter. All samplers were operated at a flow rate of 2 L/min for 24 h per sample.

### Sample preparation and analysis

The authors applied methods of sample preparation by sample extraction from the matrix (XAD-2 resin, glass fiber filter, and polyurethane foam PUF), with a mixture of 3 mL dichloromethane/acetone (1:1) followed by the chromatographic GC-MS. The chromatographic instrument was a GC MS7890A Agilent Technology Inc., with mass spectrometry 5975C Agilent Technology Inc. For the GC-MS analysis, the column used was RTX-5silMS (30 m × 0.25 mm ID × 0.25 μm). The inlet was set at 280 °C, and automatic injections of 2 μl of extracts were performed in a splitless mode. The helium carrier gas flow was set at 1 mL/min. The oven temperature program began at 40 °C and increased to 300 °C at 20 °C/min and then kept at that temperature for 15 min. The GC–MS interface was set at 280 °C. The MS detection was in a selective ion monitoring operating mode (SIM) at an electron impact energy of 70 eV. Two or three mass fragments were selected for each compound. The analysis was described more in details in the previous publication of the authors (Szewczyńska et al. 2019).

### Quality control and quality assurance

#### Calibration curve

Calibration curves in the concentrations ranging from 0.008 to 1 μg/ml were prepared for 6 phthalates. A linear regression line with the coefficient of determination (R2) higher than 0.99 was accepted. The lowest concentration that can be determined by this method for glass fiber filter and PUF was 0.066 μg/m^3^ and for XAD-2 was 0.022 μg/m^3^ at 720 l air sampling.

#### Recovery rate

Phthalate recovery from the fiber glass filter, PUF polyurethane foam, and XAD 2 Amberlite resin was studied. A certified phthalate standard solution was spiked to the filter, polyurethane foam PUF, and Amberlite resin XAD-2. Subsequently the air was passed through the sampler for 8 h, and after chromatographic analysis of the samples, the phthalate recovery was calculated. The recovery results from XAD-2 resin were at the level of 99–102%, from filters 70–98%, and from PUF 94–98%.

#### Limit of detection

The limit of detection (LOD) has been calculated on the basis of the following formula: LOD = (3.3 ∙S)/B, where *S* is the standard deviation of the blank and *B* is the slope of the calibration straight line (coefficient of direction of the calibration curve). The limit of detection for 6 phthalates was 3 ng/ml. The limit of quantification (LOQ) is 3 times the determined value of the limit of detection.

#### Blank

All of the applied materials and standards (filters, PUF and XAD-2 resin) were tested for phthalates. Therefore, the blank samples for each of these materials were taken after the clean air was passed through the sampler. The blank values were below LOD of the applied method.

## Results and discussion

Due to the differences in potential health effects on office workers, the distribution of phthalate concentrations in office rooms in the inhalable and the respirable fractions and in the gas phase was assessed. The results of different PAEs determinations in office rooms divided into empty premises directly after the renovation, renovated rooms after furnishing, and finally renovated furnished rooms already used by the employees are presented in Table 1. Table 1 shows the concentration ranges for the individual phthalates and its average values for the five rooms. In the rooms where the concentrations of phthalates were measured both in the gas phase and adsorbed on the particles, 4 different compounds were determined, i.e., DMP, DEP, DBP, and DEHP. However, each of the identified phthalate was present at different concentrations in the studied office rooms. Exemplary, none of the phthalates was detected in the gas phase in newly furnished rooms after the renovation, while DMP was not present in such room types neither in the gas phase nor in the particulates.

**Table 1:** The results of different PAE determinations in office rooms in gaseous, inhalable and respirable fraction and their total concentration

Pollutants	Test fraction/phase	Empty rooms after renovation (5 rooms) (*n* = 3)			Furnished rooms after renovation (5 rooms) (*n* = 3)			Renovated, furnished rooms after 7 months (5 rooms) (*n* = 3)		
		Ranges, μg/m^3^								
		Min	Max	Average	Min	Max	Average	Min	Max	Average
DMP	Inhalable fraction	nd	nd	nd	nd	nd	nd	nd	nd	nd
	Respirable fraction	nd	nd	nd	nd	nd	nd	bq	0.06	bq
	Gas phase	bq	4.65	2.86	nd	nd	nd	bq	0.8	0.19
DEP	Inhalable fraction	4.21	6.47	5.01	14.3	23.79	17.33	0.22	1.06	0.49
	Respirable fraction	0.24	0.92	0.56	1.5	23.25	13.60	nd	0.09	0.05
	Gas phase	bq	1.76	1.07	nd	0.25	0.08	nd	0.36	0.18
DBP	Inhalable fraction	1.26	5.89	4.55	2.36	4.75	3.15	1.46	3.36	2.08
	Respirable fraction	0.15	0.67	0.34	0.82	3.65	1.85	0.08	0.21	0.14
	Gas phase	bq	0.47	0.09	nd	nd	nd	nd	0.18	0.10
DEHP	Inhalable fraction	0.75	1.52	1.10	nd	3.7	2.22	nd	1.37	0.99
	Respirable fraction	0.14	0.33	0.20	nd	3.04	1.66	nd	0.4	0.22
	Gas phase	nd	nd	nd	nd	nd	nd	nd	nd	nd

*nd* not determined, *bq* below the quantification of the method, *DMP* dimethyl phthalate, *DEP* diethyl phthalate, *DBP* dibutyl phthalate, *DEHP* bis(2-ethylhexyl) phthalate

**Fig. 1:**
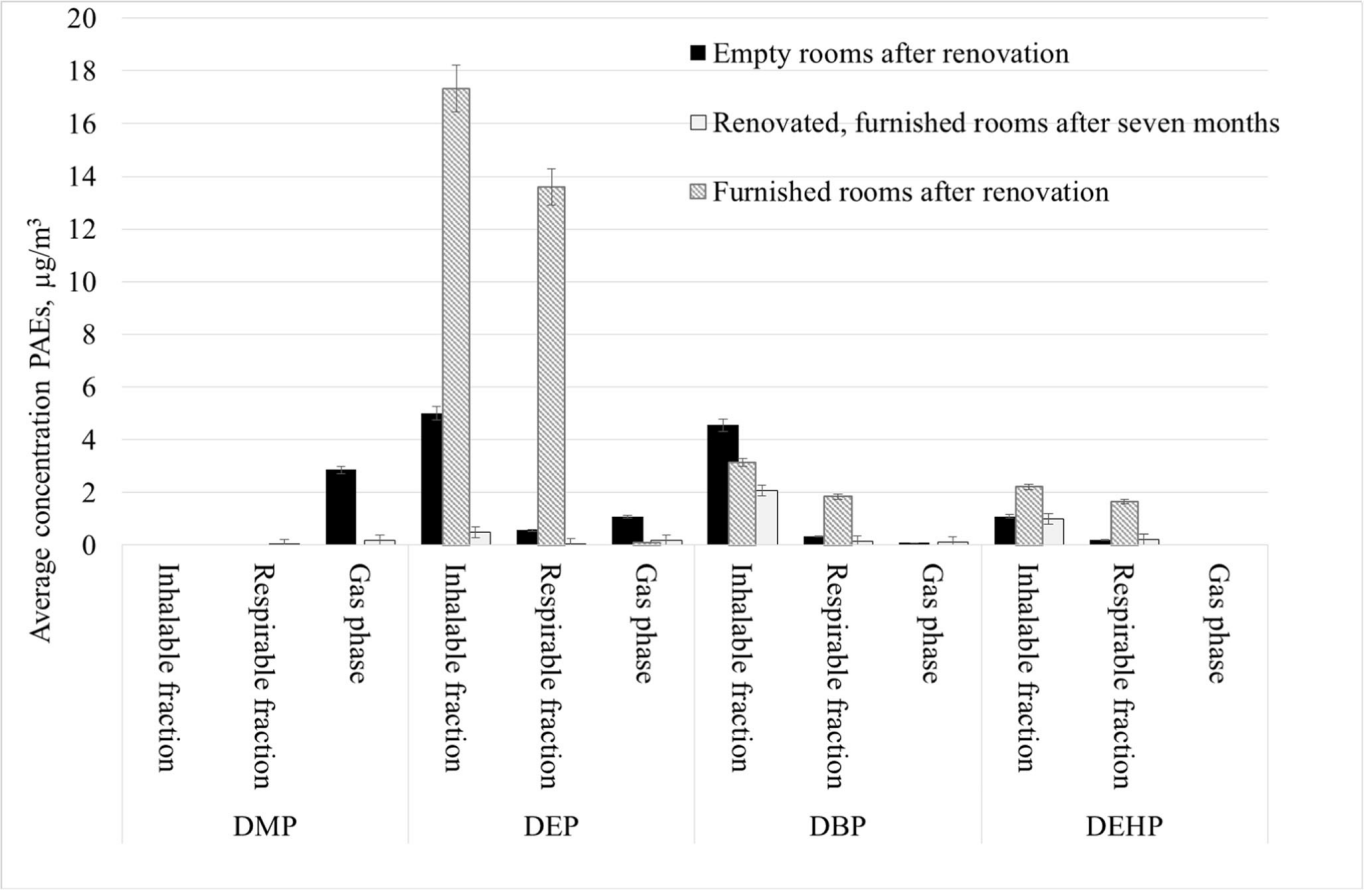
The distribution of average concentrations of particular phthalates detected in the examined rooms in the gas phase and in the inhalable and respirable fraction

Comparing the presence of phthalates in the office room on a different stage of its preparation for use by an office worker, it can be noticed that the highest average concentrations of phthalates were recorded indoors after furnishing of the renovated rooms. Figure 1 shows the distribution of the average concentrations of particular phthalates detected in the examined rooms in the gas phase and in the inhalable and respirable particulate fraction. DMP was detected in the gas phase in empty rooms after the renovation (mean concentration was 2.8 μg/m^3^) and in premises where measurements were carried out 7 months after the renovation (mean concentration of 0.19 μg/m^3^). DEP and DBP were determined both in the gas phase and in the inhalable and the respirable particulates fractions in all of the investigated rooms, while DEHP was determined in all of these rooms but only in the particulate fractions. In the inhalable fraction, the highest mean concentration of DEHP was determined in furnished rooms after the renovation and amounted to 2.2 μg/m^3^ and in the respirable fraction to 1.7 μg/m^3^. Thus, DEHP detected in the respirable fraction penetrating into the gas exchange area represents 75% of the analyte determined in the inhalable fraction.

**Fig. 2:**
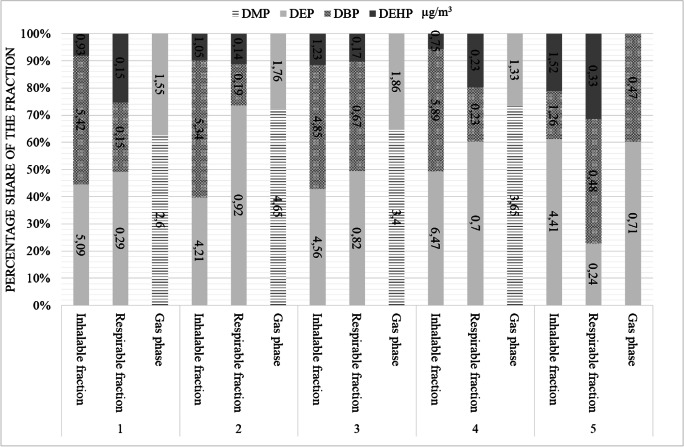
The concentration distribution individual phthalates determined in five empty rooms after renovation and their percentage ratio

The research started in five office rooms directly after their renovation, which covered wall painting, replacing the windows and floor exchange. The concentration distribution of the individual phthalates determined in these rooms (1–5) and their percentage share in the particulate and gas phase is shown in Fig. 2. The highest concentrations of volatile PAEs in the gas phase (DMP and DEP) were 4.65 and 1.86 μg/m^3^. In the respirable and the inhalable fraction, DEP, DBP, and DEHP were determined. In this case, total average phthalates in the gaseous phase accounted for 37.7% of the total average phthalates determined in the inhalable fraction.

**Fig. 3:**
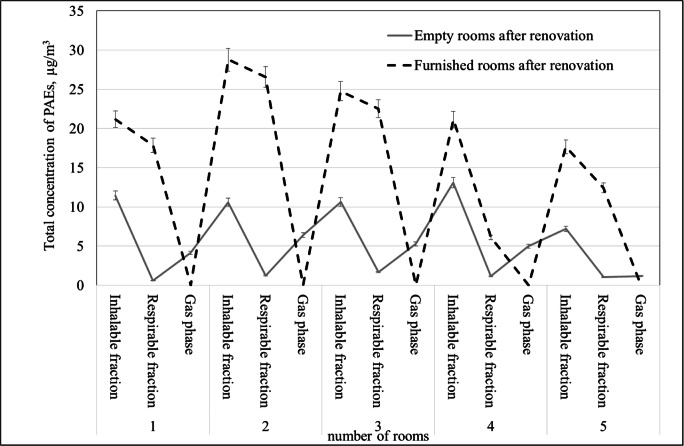
Comparison of total phthalate concentrations in the gas phase and in the particulates fractions in empty rooms after the renovation and renovated furnished rooms

The next stage of the research included phthalates determination in the same rooms after their furnishing. Room furnishing with office furniture and computer equipment increased the concentration of phthalates in both fractions of particulates in these rooms. Comparison of the total concentration of phthalates determined in empty and furnished rooms is presented in Fig. 3. The highest concentrations of the identified phthalates both in the respirable (26.56 μg/m^3^) and the inhalable (28.78 μg/m^3^) fractions were determined in three renovated rooms after their furnishing. The average total concentration of phthalates from all the furnished rooms detected in the inhalable and respirable fractions was 22.7 μg/m^3^ and 17.1 μg/m^3^, respectively. These concentrations were higher 2.1 times for inhalable phthalates and 15 times for respirable phthalates than the average total PAEs concentration in empty rooms. Such results indicated that in the office rooms, phthalates may be released not only from the materials used for renovation but also from the furniture, carpets, and the office equipment. It should be noted that the rooms selected for measurements were furnished with desks with plastic binders and containers for paper utensils, cabinets, chairs with plastic elements, and electronic equipment.

**Fig. 4:**
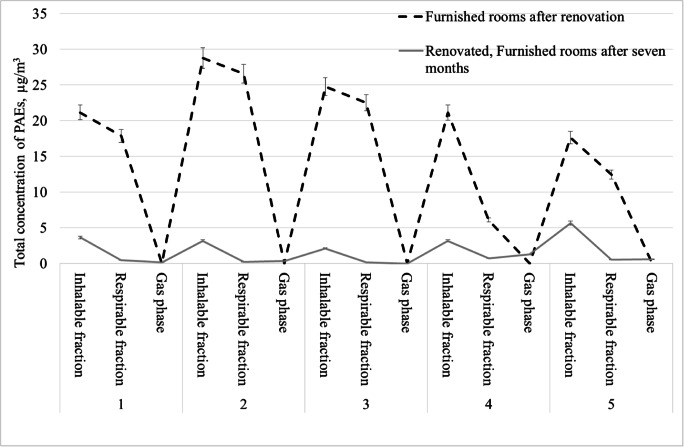
Comparison of total phthalate concentrations in the gas phase and in the particulates fractions in renovated rooms after furnishing and furnished rooms measured 7 month after the renovation

**Fig. 5:**
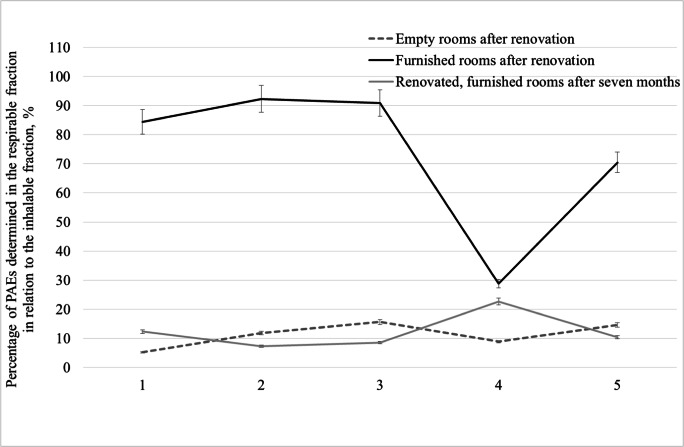
Percentage of phthalates in the respirable fraction in relation to phthalates in the inhalable fraction

Measurements of total phthalates concentrations in office premises that were being used for 7 months after their renovation and furnishing showed significantly lower phthalate concentrations (Fig.4). Their highest total concentration in the inhalable fraction was 5.68 μg/m^3^, whereas previously in the same room (number 5) directly after its furnishing, the concentration was 17.6 μg/m^3^. These rooms often had an air conditioning and were ventilated by workers, thus probably contributed to a decrease in the concentration of the tested compounds. Comparing the average results for the sum of phthalates in the inhalable fraction, the concentrations of phthalates in renovated rooms after 7 months of their use decreased by 84% compared with the concentration of phthalates in freshly furnished rooms and by 68% compared with the concentration of phthalates in empty renovated rooms. A similar dependency was observed by Song and col. (Song et al. 2015) suggesting that PAE concentrations in offices decreased by 50% 2 years after decorating student rooms. In conclusion, the total concentration of phthalates determined in the respirable fraction in furnished rooms constituted as much as 92% of phthalates determined in the inhalable particulate fraction. In empty rooms immediately after the renovation and rooms furnished and used for 7 months by the employees, phthalates determined in the respirable fraction did not exceed 25% of these compounds in the inhalable fraction (Fig. 5). It should be noted that the respirable fraction contained DBP and DEHP, which are classified as endocrine disruptors (category 1) and reprotoxic substances (hazard category 1B). The phthalates adsorbed on the respirable particulates fraction can be transported by air and inhaled into the human respiratory system. This poses a potential health risk for office workers because the respirable fraction is deposited in the gas exchange area.

Workers’ exposure to low concentrations of phthalates (at microgram levels) in a cubic meter of air should not be excluded from the risk assessment because these compounds acting as endocrine disruptors can cause the increase of many reproductive disorders in men and women (Rutkowska et al. 2015). In men, they can cause a decrease in sperm count, testicular failure, a decrease in testosterone concentration in the blood, and the occurrence of benign testicular tumors. Some phthalate esters inhibit steroid genesis in Leydig’s interstitial cells in male gonads (Queiroz and Waissmann 2006). In women, it can cause premature breast adolescence, as well as liver, kidney, and heart damage. Phthalates, like other plastic components, can penetrate the placental barrier and can be dangerous to the unborn child. These compounds are also considered to be respiratory allergens to the effect of asthma, and they reduce cognitive abilities, e.g., spatial orientation, memory, perception, excessive motor activity, and behavioral disorders (aggression), and reduce social contacts (D’Amato et al. 2015).

## Conclusions

Since phthalates, acting as endocrine disruptors, can cause many reproductive disorders in men and women, their presence in the air should not be underestimated. The measurements carried out in the presented paper indicate a variation in the concentrations of phthalates at the workstations covered by the study.

As it results from the presented research, the phthalates were determined in the air in the gaseous phase and deposited on the particulates in the inhalable and respirable fraction. The average concentration of total PAEs in gas phase and particulate fraction ranged from 4.4 to 39.8 μg/m^3^. Of all the PAEs detected, DEHP, DBP, and DEP were present in most of the studied office rooms. The gas fraction was dominated by DMP, DEP, and the inhalable fraction by DBP and DEHP which are classified as endocrine active substances. The phthalates in the respirable fraction represent a maximum of 92% of the phthalates determined in the inhalable fraction. The respirable fraction is the part of the inhaled airborne particles that can pass into the gas exchange region in the lungs, and therefore, it may pose higher potential threat to workers. The highest concentrations of phthalates were marked in the office rooms after they were equipped with furniture and office equipment. It is therefore advisable to pay attention to adequate ventilation of the rooms, which will contribute to the reduction of the concentration of harmful substances and improve the comfort of work.

It can be noticed that the total concentration of phthalates in the office rooms measured 7 months after their renovation in the inhalable fraction decreased on average by 76% compared with that in newly renovated rooms.
